# ER–mitochondria signaling in Parkinson’s disease

**DOI:** 10.1038/s41419-017-0079-3

**Published:** 2018-03-01

**Authors:** Patricia Gómez-Suaga, José M Bravo-San Pedro, Rosa A. González-Polo, José M. Fuentes, Mireia Niso-Santano

**Affiliations:** 1 Department of Basic and Clinical Neurosciences, Institute of Psychiatry, Psychology and Neuroscience, King’s College London, London, SE5 9RX UK; 2 Equipe 11 Labellisée Ligue contre le Cancer, Centre de Recherche des Cordeliers, 75006 Paris, France; 3 INSERM U1138, 75006 Paris, France; 4 Université Paris Descartes/Paris V, Sorbonne Paris Cité, 75006 Paris, France; 5 Université Pierre et Marie Curie/Paris VI, 75006 Paris, France; 6 Gustave Roussy Comprehensive Cancer Institute, 94805 Villejuif, France; 7 Centro de Investigación Biomédica en Red en Enfermedades Neurodegenerativas (CIBERNED), 18100 Granada, Spain; 8 Facultad de Enfermería y Terapia Ocupacional, Universidad de Extremadura. Avda. De la Universidad S/N, C.P, 10003 Cáceres, Spain

## Abstract

Mitochondria form close physical contacts with a specialized domain of the endoplasmic reticulum (ER), known as the mitochondria-associated membrane (MAM). This association constitutes a key signaling hub to regulate several fundamental cellular processes. Alterations in ER–mitochondria signaling have pleiotropic effects on a variety of intracellular events resulting in mitochondrial damage, Ca^2+^ dyshomeostasis, ER stress and defects in lipid metabolism and autophagy. Intriguingly, many of these cellular processes are perturbed in neurodegenerative diseases. Furthermore, increasing evidence highlights that ER–mitochondria signaling contributes to these diseases, including Parkinson’s disease (PD). PD is the second most common neurodegenerative disorder, for which effective mechanism-based treatments remain elusive. Several PD-related proteins localize at mitochondria or MAM and have been shown to participate in ER–mitochondria signaling regulation. Likewise, PD-related mutations have been shown to damage this signaling. Could ER–mitochondria associations be the link between pathogenic mechanisms involved in PD, providing a common mechanism? Would this provide a pharmacological target for treating this devastating disease?

In this review, we aim to summarize the current knowledge of ER–mitochondria signaling and the recent evidence concerning damage to this signaling in PD.

## Facts

- Endoplasmic reticulum (ER) and mitochondria form close associations that constitute key signaling hubs to regulate many cellular processes.
- ER–mitochondria contacts regulate many different pathways, which are damaged in Parkinson’s disease (PD).
- ER–mitochondria associations are altered in PD.

## Open questions

- Are ER–mitochondria associations disrupted or upregulated upon PD-related insults?
- Is ER–mitochondria signaling damage the common link among the different pathways involved in PD?
- What are the molecular mechanisms implicated in PD-related protein damage to ER–mitochondria associations?
- Do other PD-related proteins alter ER–mitochondria signaling?
- Is ER–mitochondria signaling also damaged in sporadic PD?
- Can ER–mitochondria signaling be targeted therapeutically?

## Introduction

Parkinson’s disease (PD) is the most common movement disorder and the second most common neurodegenerative disease after Alzheimer’s disease (AD). PD patients typically experience difficulties with slowness of movements (bradykinesia), involuntary shaking (tremor), increased resistance to passive movement (rigidity) and postural instability. The cardinal motor symptoms of PD are attributable to the progressive degeneration of dopaminergic neurons in the *pars compacta* of the *substantia nigra* (SNpc DA). PD is also characterized by the presence of intraneuronal proteinaceous inclusions called Lewy bodies (LB) and abnormal dystrophic neuronal processes termed Lewy neurites in the surviving neurons^1^.

Although most cases are sporadic, mutations in several genes, the *PARK* loci, have been unequivocally shown to cause familial parkinsonism in 5–10% of cases. Importantly, the phenotypes of both the sporadic and familial forms are essentially indistinguishable, implying that they might share common underlying mechanisms. Mutations in three genes, *SNCA* (best known as α-synuclein), *LRRK2* (Leucine-rich repeat kinase 2), and *VPS35* (Vacuolar protein sorting-associated protein 35), are known to cause a dominant form of PD, whereas mutations in *PARK2* (parkin RBR E3 ubiquitin protein ligase, best known as Parkin), *PINK1* (PTEN-induced putative kinase 1), and *PARK7* (Parkinsonism associated deglycase, best known as DJ-1) cause recessive-inherited forms of the disease^2^. The discovery of such monogenic forms during the last two decades has significantly advanced our understanding of the pathogenic mechanisms involved in PD, as it allows for the generation of animal and cellular models carrying the mutant gene. Thus, although the precise mechanisms underlying neuronal death in PD remain to be determined, damage to a plethora of cellular processes has been widely reported. These include alterations in Ca^2+^ homeostasis, cellular proteostasis, axonal transport, mitochondrial function, and neuroinflammation^3^. Consequently, one of the difficulties in deciphering PD-related toxicity consists of linking these apparently diverse pathological changes to a common disease pathway.

Recently, several indications have argued in favor of the possibility that perturbations in the ER–mitochondrial network have an important role in the pathogenesis of PD^4,5^. Indeed, ER–mitochondria communication has been demonstrated to be altered in several neurodegenerative diseases, including PD^4^. This review is mainly devoted to discussing the evidence that ER–mitochondria signaling dysfunction may have a role in PD pathogenesis.

## Endoplasmic reticulum–mitochondria associations

In the eukaryotic cell, communication and cooperation between the different membrane-bound organelles must take place to integrate cellular physiology. This integration depends upon effective crosstalk and one way in which this is achieved is through direct membrane contact. Thus, proper endoplasmic reticulum (ER)–mitochondria communication requires the formation of specialized membrane microdomains at the contact sites, defining short distances between membranes to connect them^6^. The ER and mitochondria association is the most studied and the first described inter-organelle contact^7^. The ER is closely opposed to 5–20% of the mitochondrial surface. The ER domain specialized in this association is known as mitochondria-associated membranes (MAMs) and can be smooth or ribosome-containing rough ER membranes^8,9^.

### ER–mitochondria tethering complexes

**Fig. 1:**
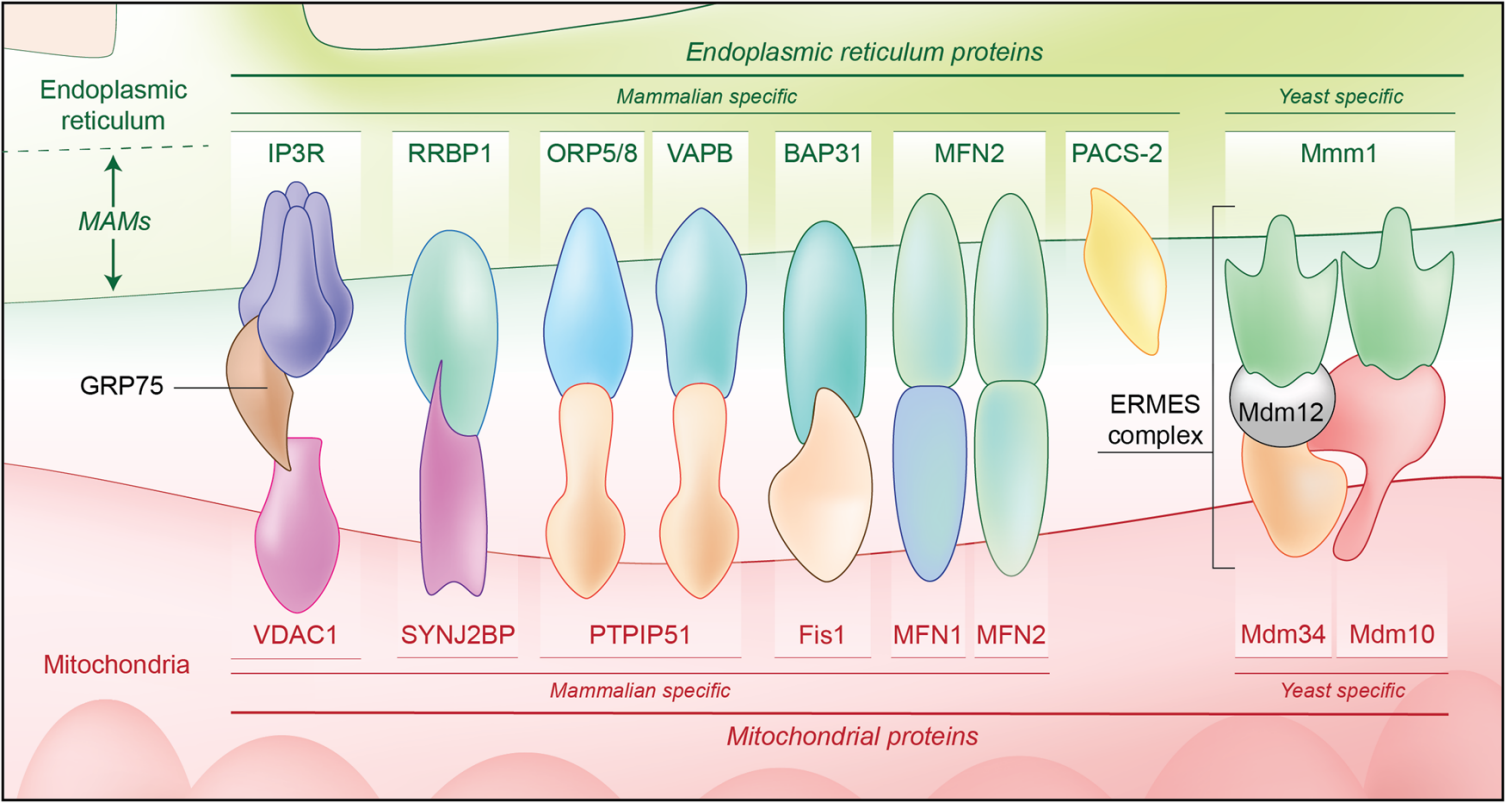
Endoplasmic reticulum–mitochondria tethering complexes Multiple structures that tether mitochondria with the mitochondria-associated membranes (MAMs) of endoplasmic reticulum (ER) have been described. Inositol 1,4,5-trisphosphate receptor (IP3R) and voltage-dependent anion channel (VDAC1) interact via GRP75. Synaptojanin 2 binding protein (SYNJ2BP) interacts with the ribosome-binding protein 1 (RRBP1). The outer mitochondrial protein tyrosine phosphatase-interacting protein 51 (PTPIP51) interacts with vesicle-associated membrane proteins-associated protein B (VAPB) or oxysterol-binding protein-related proteins (ORP5/8) at the ER. B-cell receptor associated protein 31 (BAP31) binds to mitochondrial fission 1 protein (Fis1). ER-located mitofusin 2 (MFN2) interacts with mitochondrial MFN1/MFN2. Other proteins, such the ER sorting molecule phosphofurin acidic cluster sorting protein-2 (PACS-2), have been involved in ER–mitochondria association integrity. Yeasts specific proteins have also been described: the ER–mitochondria encounter structure (ERMES) complex, composed of four proteins: the outer mitochondrial membrane proteins Mdm10 and Mdm34, the ER protein Mmm1, and the cytosolic protein Mdm12

The presence of structures that appear to tether the two organelles has been observed by electron microscopy in many different cell types^4,6,10–13^ (Fig. 1). Early studies revealed the proteinaceous nature of the tethers between the two membranes^6,14^. Studies in yeast revealed the presence of a protein complex, known as ERMES (ER–mitochondria encounter structure)^15^. However, no mammalian orthologues of ERMES proteins have been identified yet; on the contrary, several different protein complexes have been proposed as ER–mitochondria tethers^16^. One of these complexes is based on the interaction between the ER Ca^2+^ channel IP3R (inositol 1,4,5-trisphosphate receptor) and the OMM VDAC1 (voltage-dependent anion channel 1), that is the major mitochondrial Ca^2+^ transport channel, in a ternary binding complex with the mitochondrial chaperone GRP75 (glucose-regulated protein 75)^17^. The ER sorting molecule PACS-2 (phosphofurin acidic cluster sorting protein-2) has also been shown to be involved in ER–mitochondria associations^18^. Similarly, the interaction between the ER protein Bap31 (B-cell receptor associated protein 31) and the mitochondrial fission protein Fis1 has been shown to bridge the mitochondria and the ER and promote apoptosis^19^.

Another MAM protein, mitofusin 2 (MFN2), has also been proposed as a tethering complex by establishing homo- and heterotypic interactions with mitochondrial MFN1/2^20^. However, the role of MFN2 as an ER–mitochondria tether has been challenged as several recent studies from different laboratories have now shown that loss of MFN2 leads to an increase and not a decrease in ER–mitochondria contacts^21–24^. Thus, whether MFN2 is functionally involved in ER–mitochondria tethering remains to be resolved.

The vesicle-associated membrane proteins-associated proteins (VAPs) are integral ER membrane proteins, which interact with a plethora of proteins to mediate associations between the ER and other membranes^25^. These include mitochondria but also peroxisomes, the Golgi, the plasma membrane, and the endo-lysosome compartment^26–30^. Mammals have two homologous VAP proteins, VAPA and VAPB, which share 76% similar or identical amino acid residues^31^. VAPB binds to the OMM protein, PTPIP51 (protein tyrosine phosphatase-interacting protein 51) to tether ER with mitochondria^10,32^. Thus, manipulating VAPB or PTPIP51 expression has been shown to induce appropriate changes in ER–mitochondria contacts^10,33^. An amyotrophic lateral sclerosis (ALS)-related VAPB mutation has been shown to increase the PTPIP51-dependent interaction between the ER and mitochondria^32^. Regarding PD, a recent study showed that the PD-related protein α-synuclein interacts with VAPB, decreasing the VAPB-PTPIP51 interaction (see below)^34^.

In addition, PTPIP51 can interact with the oxysterol-binding protein-related proteins ORP5 and ORP8 to tether mitochondria to ER^13^. ORP proteins have been thought to have a role as sterol sensor or transport proteins^35,36^. Recently, PTPIP51 has been involved in regulating the interaction of mitochondria with the sarcoplasmic reticulum, a specialized type of ER, in cardiac function^37^.

Although rough ER–mitochondria contacts have long been observed by electron microscopy^6,38–40^, the above mentioned tethers appear to be specific for ribosome-excluded mitochondria-smooth ER contacts. Interestingly, a recent study has identified novel protein candidates that reside at rough ER–mitochondria contact sites, the OMM protein SYNJ2BP (synaptojanin 2 binding protein), which interacts with the ER protein RRBP1 (ribosome- binding protein 1)^41^.

### Cellular functions regulated by ER–mitochondria signaling

**Fig. 2:**
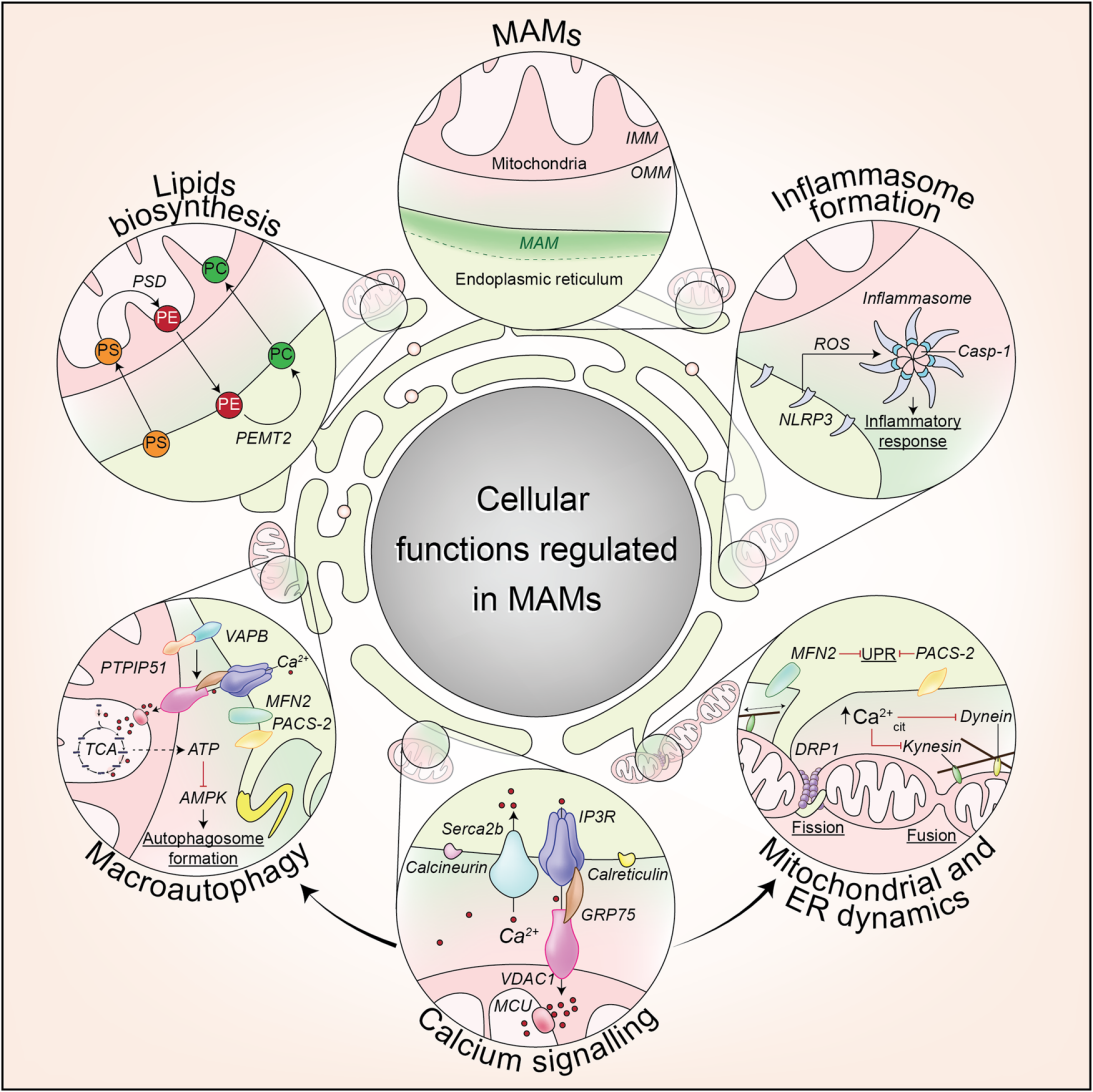
Endoplasmic reticulum–mitochondria signaling function The interaction between mitochondria and the MAMs of ER has been linked with different cellular functions, including inflammasome formation; calcium (Ca^2+^) signaling, mitochondrial and ER dynamics, autophagy and lipid biosynthesis. AMPK AMP-activated protein kinase, Casp-1 caspase-1, DRP1 dynamin-related protein 1, GRP75 glucose-regulated protein 75, IMM inner mitochondrial membrane, IP3R inositol 145-trisphosphate receptor, MCU mitochondrial calcium uniporter, MFN2 mitofusin 2, NLRP3 NLR family pyrin domain-containing 3, OMM outer mitochondrial membrane, PACS-2 phosphofurin acidic cluster sorting protein-2, PC phosphatidylcholine, PE phosphatidylethanolamine, PEMT2 phosphatidylethanolamine N-methyltransferase 2, PS phosphatidylserine, PSD phosphatidylserine decarboxylases, PTPIP51 protein tyrosine phosphatase-interacting protein 51, ROS radical oxygen species, TCA tricarboxylic acid cycle, UPR unfolded protein response, VAPB vesicle-associated membrane proteins-associated protein B, VDAC1 voltage-dependent anion channel 1

ER–mitochondria contacts are historically linked to lipid metabolism and Ca^2+^ signaling^8,9^. Nevertheless, further studies have revealed additional roles for ER–mitochondria signaling in a variety of processes ranging from intracellular trafficking of mitochondria and ER to cell survival, energy metabolism, protein folding and autophagy^11,33,42–46^. Here, we will give a brief description of the most important ER–mitochondria signaling functions (Fig. 2).

#### ER–mitochondria contacts serve as a platform for lipid biosynthesis

ER–mitochondria contacts mediate shuttling of lipids between the two organelles, which is necessary for the synthesis of certain lipids such as phosphatidylcholine (PC)^47^. In fact, this role in the transfer of phospholipids was the first function attributed to ER–mitochondria associations^9^. For this, phosphatidylserine (PS) is first synthesized in the MAM by the PS synthase 1 and 2; then it is transferred to mitochondria where a decarboxylase (PSD) converts it to phosphatidylethanolamine (PE); PE is crucial for the maintenance of mitochondrial tubular morphology and therefore for mitochondrial functions^48,49^. PE can be transferred back to the ER, where phosphatidylethanolamine N-methyltransferase-2 (PEMT2) converts it into PC^47^.

#### Inflammasome formation

Inflammation is a tightly regulated response of the innate immune system to combat infection or tissue injury and it involves the production of pro-inflammatory cytokines. One of the innate immunity sensors that can orchestrate inflammatory response, by secreting pro-inflammatory cytokines IL-1β and IL-18, are cytosolic multiprotein complexes termed inflammasomes. Upon its activation, the inflammasome complex mediates activation of caspase-1, which represents a crucial step in the secretion of the previously mentioned cytokines and consequently drives the inflammatory response. The NLRP3 inflammasome is the most studied inflammasome and it is formed after the oligomerization of NLRP3 and subsequent recruitment of apoptosis-associated Speck-like protein with a caspase-recruitment domain and pro-caspase-1^50^. One class of these is the NOD-like receptors (NLRs), which sense abnormal cytosolic changes. Upon activation by ROS, some NLRs, including NLRP3, form multiprotein complexes, which redistribute to MAM to activate the inflammasome^51^.

#### ER–mitochondria contact sites are crucial for efficient cellular Ca^2+^ handling and Ca^2+^-regulated processes

Early studies by Rizzuto and co-workers showed that ER–mitochondria associations mediate Ca^2+^ transfer from ER to mitochondria^8,17,52^. These studies demonstrated that the close apposition between the two organelles at contact sites allows the formation of hotspots that meet the low affinity threshold of mitochondrial Ca^2+^ uptake mechanisms. Consequently, MAM are enriched in proteins associated with Ca^2+^ handling such as channels and chaperones^53^. As previously mentioned, the ER Ca^2+^ channel IP3R contacts VDAC1 through the molecular chaperone GRP75, mediating the Ca^2+^ transfer from the ER to mitochondria^17^. In addition, chaperones located at MAM, like calnexin and calreticulin, can interact with the IP3R and the ER Ca^2+^ transport ATPase SERCA2b to regulate Ca^2+^ signaling^53^. Although the ER is the major Ca^2+^ store, mitochondria are also an important Ca^2+^ reserve, especially in neurons, so the Ca^2+^ transfer between them is crucial for the maintenance of cellular Ca^2+^ homeostasis^54^.

One of the first roles assigned to the mitochondrial Ca^2+^ uptake from the ER was the regulation of mitochondrial oxidative metabolism^55^. Mitochondrial activities are driven in a Ca^2+^-dependent manner as three dehydrogenases in the tricarboxylic acid cycle (pyruvate-, α-ketoglutarate-, and isocitrate-dehydrogenases) as well as mitochondrial FAD-glycerol phosphate dehydrogenase are activated by Ca^2+^^55^. However, prolonged mitochondrial Ca^2+^ overload compromises mitochondrial function by causing a transient collapse of the mitochondrial membrane potential, leading to necrosis or apoptosis^54,56^.

Regarding their role in Ca^2+^ homeostasis and bioenergetics^57–59^, ER–mitochondria associations has been demonstrated to regulate macroautophagy^33^. Macroautophagy, (hereafter called autophagy), is a lysosomal mechanism of degradation that can be activated during metabolic energy stress, a condition in which the process promotes the recycling of intracellular contents to produce metabolic intermediates^60^. As mentioned above, mitochondrial Ca^2+^ uptake through ER–mitochondria contact sites is necessary for ATP production. Consequently, blocking Ca^2+^ transfer to mitochondria was shown to stimulate autophagy as a physiological response of the cell to the altered bioenergetics^57^. Recently the ER–mitochondria tethering complex VAPB-PTPIP51 was shown to modulate autophagy, involveing their role in mediating IP3R-mediated delivery of Ca^2+^ from ER stores to mitochondria^33^.

Furthermore, ER–mitochondria contacts serve as membrane source for autophagosome formation^44^. During the autophagy process, specialized double-membrane vesicles, known as autophagosomes, are formed. Autophagosome formation starts with an initial isolation membrane, known as the phagophore, which expands by de novo membrane synthesis and recruitment of lipids and proteins from different membrane sources. Then, the autophagosome engulfs this material for degradation and fuses with the endosomal–lysosomal system where the cargo is degraded and recycled^60^. Several organelles have been proposed to provide the nucleation site and to contribute to the formation and expansion of the autophagosomal membrane. The involvement of ER and mitochondria to this process has been extensively reported, including the ER–mitochondria contact sites^61^. Hence, upon autophagy induction, several pro-autophagic proteins relocalize to MAMs to initiate autophagosome formation^44,62–64^.

#### Regulation of ER and mitochondrial dynamics and homeostasis

ER–mitochondria associations are also important in the movement of both organelles. ER and mitochondria are dynamic organelles transported on cytoskeletal elements. Importantly, ER–mitochondria contacts have been shown to be maintained while both organelles are moving^42^. This transport involves specialized molecular machinery, as molecular motors such as dynein and kinesin, which are tightly regulated by Ca^2+^ sensors^65–67^. Hence, a rise in cytosolic Ca^2+^ concentration has been shown to produce an arrest of the movements of both organelles^68–70^.

Besides participation in mitochondrial motility, MAMs also participate in the regulation of mitochondrial morphology and biogenesis, which is maintained by the balance between fission and fusion events^71^. Accordingly, MAM is also enriched in proteins related to the control of mitochondrial fission^11^ and dynamics^72^. Indeed, mitochondrial fission occurs at positions where ER tubules contact and constrict mitochondria^11^. These constrictions facilitate the recruitment of DRP1 (dynamin-related protein), a major player in mitochondrial fission^73^. In addition, ER–mitochondria contacts have a key role during mitophagy, the selective degradation of mitochondria through the autophagy pathway^74^. During hypoxia, the interaction between the OMM protein FUNDC1 and the ER chaperone calnexin gets disrupted and, as mitophagy proceeds, FUNDC1 preferably recruits DNM1L/DRP1 to drive mitochondrial fission promoting mitophagy^64^. Importantly, a recent study showed the recruitment into MAM of the PD-related proteins PINK1 and Parkin with downstream effects on ER–mitochondria associations and mitophagy, as explained in detail later in this review^62^.

The homeostasis of the ER can be altered by several conditions including Ca^2+^ depletion from its lumen and oxidative stress. These perturbations result in disruption of the folding process in the ER, leading to the accumulation of misfolded/unfolded proteins and ER stress. ER stress then activates the unfolded protein response (UPR), a complex signal-transduction pathway that mediates cellular adaptation to restore ER homeostasis^75^. A number of ER protein folding chaperones are present in MAM and alterations to ER–mitochondria signaling is linked to UPR^53^ For example, the structural uncoupling of ER from mitochondria by depletion of PACS-2 or MFN2 was shown to induce ER stress and the UPR^18,76,77^. Likewise, VAPB also has roles in the UPR^78,79^.

In consequence, disease-related insults that cause an abnormal tightening or loosening of ER–mitochondria contacts are predicted to be detrimental to cells. Therefore, it is not surprising that alterations in the ER–mitochondria associations have been described in several diseases, including a number of neurodegenerative diseases^4,80–82^.

## ER–mitochondria signaling in neurodegeneration

Neurodegenerative diseases including PD, AD, and ALS/FTD (frontotemporal dementia) share several obvious features: they are characterized by progressive nervous system dysfunction, affect millions of people worldwide and there is still no cure for any of them. Furthermore, despite affecting different brain regions PD, AD, and ALS/FTD also share other characteristics suggesting that common cellular processes may converge^4^.

**Fig. 3:**
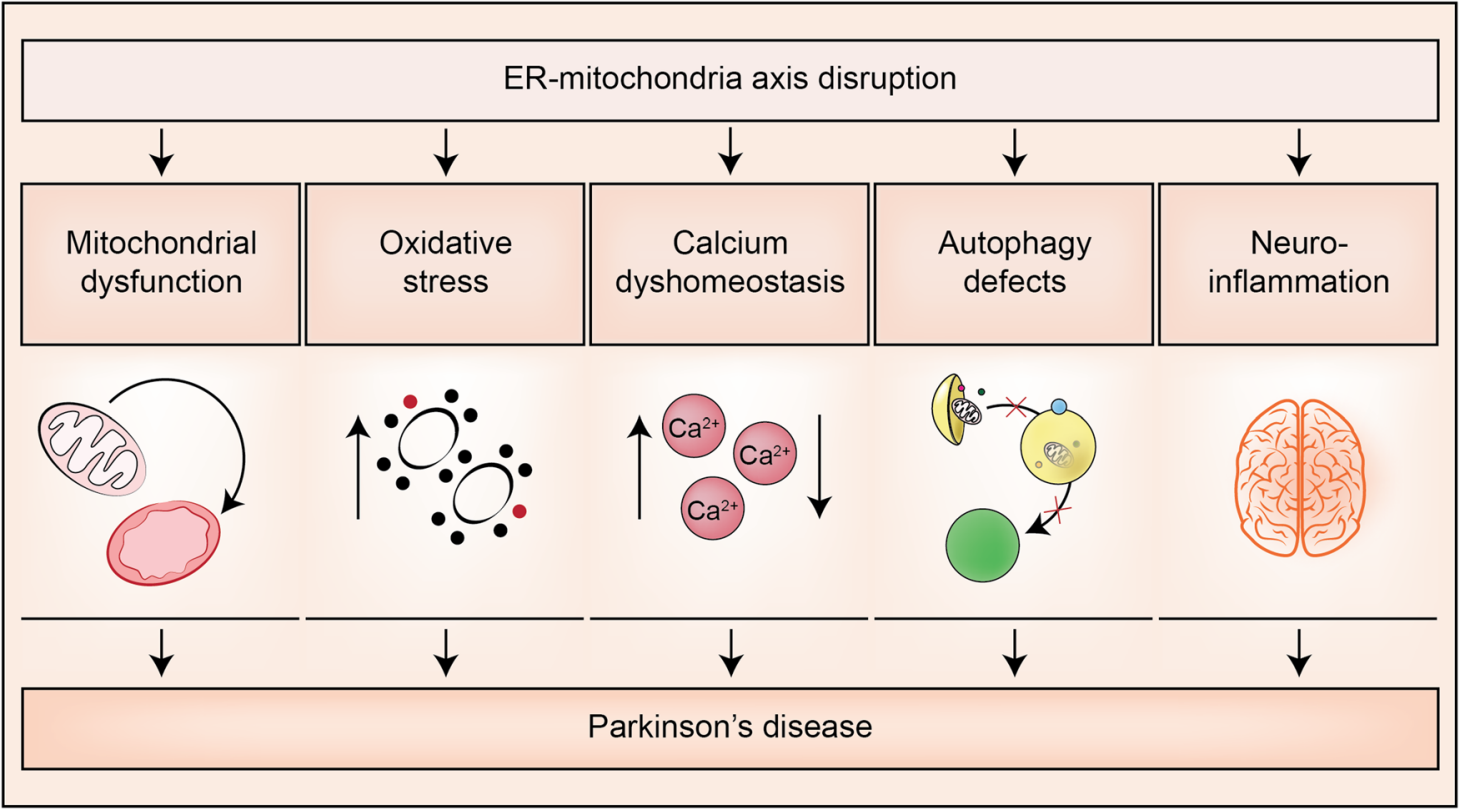
Proposed model for endoplasmic reticulum–mitochondria signaling in PD ER–mitochondrial axis appears to be essential for the healthy neurons. Conversely, the disruption of this interaction may involve the develop of some processes as: mitochondrial dysfunction, induction of oxidative stress, calcium (Ca^2+^) dyshomeostasis, autophagy defects or neuroinflammation, which induce neuronal damage and trigger neurodegenerative diseases as PD

Thus, whilst the precise mechanisms remain to be determined, a variety of cellular processes are damaged in all of them, including Ca^2+^ dysregulation, defects in axonal transport, neuroinflammation, loss of cellular proteostasis and mitochondrial dysfunction^83–88^ (Fig. 3). Remarkably, ER–mitochondria associations, regulates all of those processes. The findings that alterations in ER–mitochondria associations occur in neurodegenerative diseases have given rise to the hypothesis that damaged ER–mitochondria signaling is a common potential therapeutic target amongst distinct age-dependent neurodegenerative disorders.

This review focuses on current knowledge of ER–mitochondria signaling in PD. The roles for MAM in other neurodegenerative diseases will be addressed in other chapters of this special issue and have also been recently reviewed in ref.^4^.

### PD and ER–mitochondria signaling

#### What causes SNpc DA neurons to die in PD?

This is one of the major unresolved questions that has puzzled researchers for many years. Although the mechanisms responsible for the preferential loss of SNpc DA neurons in PD are still a debate, several studies show evidence for a role of Ca^2+^ signaling in PD pathogenesis^89,90^. Surmeier et al.^91^ proposed that the selective vulnerability of SNpc DA neurons relies on their unusual physiological characteristic; adult SNpc DA neurons are autonomously active, this means that they generate action potentials in the absence of conventional synaptic input^92^. This activity is sustained by their specific voltage-dependent L-type Ca^2+^ channels, the Cav1.3 channels, which allow Ca^2+^ influx that contributes to the membrane potential threshold underlying autonomous pacemaking, causing sustained increases in cytosolic Ca^2+^ concentrations in these cells^93,94^. As the spatiotemporal pattern of Ca^2+^ signaling is crucial for the specificity of cellular responses, Ca^2+^ must be under a tight homeostatic control which requires energy. Consequently, SNpc DA neurons experience a high ATP demand that compromises mitochondrial function and increases the production of reactive oxygen species. These events would have detrimental effects on neuronal viability and could amplify the effects of environmental factors or genetic defects^89^.

Likewise, both mitochondria and ER have been widely linked to pathogenesis in PD^95,96^. Toxins that nominally target mitochondria have been shown to induce dopamine cell degeneration^96^. Furthermore, several studies have evidenced a potential link between proteins known to cause familial PD and defects in mitochondria^96^.

Apart from a Ca^2+^ store, the ER is crucial in cellular proteostasis as it is responsible for the production, delivery and degradation of proteins^75^. Loss of proteostasis is part of the pathogenesis of many neurodegenerative diseases, including PD^97^. As previously mentioned, one of the hallmarks of PD is the formation of LBs, which reflects a deficiency in proteostasis that is accompanied by signs of ER stress^98^. As a mechanism for proteostasis, autophagy has a crucial role in the maintenance of protein and organelle homeostasis in the axons, especially in SNpc neurons, which pose an enormous axonal field^99^. In fact, many studies support a role for autophagy in PD^100^.

Given its essential role in the above mentioned cellular processes, perturbations in ER–mitochondria associations are expected to be especially detrimental to SNc DA neurons. Several familial PD-related proteins have been shown to cause alterations in ER–mitochondria signaling^34,62,101–106^. However, there is not yet a consensus on the effects of these different PD-associated insults, nor on the mechanisms leading to altered ER–mitochondria associations, which are still unclear. Likewise, whether the disease begins with the dysfunction of ER–mitochondria signaling remains elusive.

**Fig. 4:**
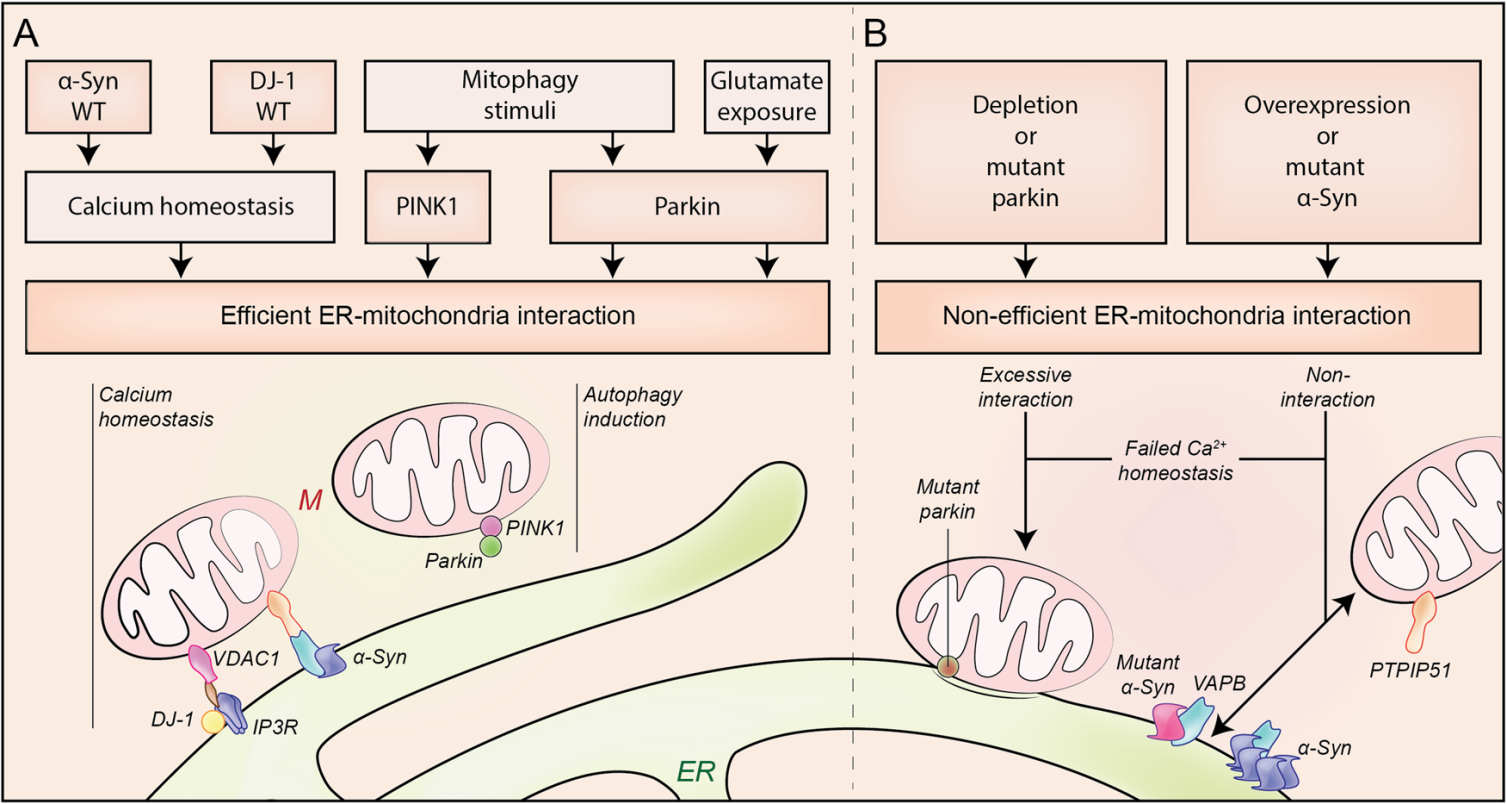
Modulators of ER–mitochondria associations Several PD-associated proteins localize at the ER–mitochondrial (M) axis and have been shown to participate in ER–mitochondria signaling regulation. Proteins such as α-synuclein (α-syn), DJ-1, PINK1 (PTEN-induced putative kinase 1), or Parkin have an important role in the preservation of healthy cells by regulation of calcium (Ca^2+^) homeostasis and the autophagic responses under different stimulus (**a**). Dysfunction of these PD-associated proteins leads to a non-efficient interaction between ER and mitochondria that triggers cell damage (**b**). IP3R inositol 145-trisphosphate receptor, PTPIP51 protein tyrosine phosphatase-interacting protein 51, VAPB VAMP-associated protein B, VDAC1 voltage-dependent anion channel 1

Despite the plausible role of ER–mitochondria signaling in PD, ER–mitochondria contacts are poorly characterized in neurons and the exact role of these associations in neuronal (patho)physiology also remains unclear. Several studies have confirmed the presence of ER–mitochondria contacts in neurons^107–110^. The presence of these contacts at synapses suggests a role in synaptic activity. In fact, in mouse respiratory neurons, ER–mitochondria axis-mediated Ca^2+^ handling was shown to determine exocytosis and synaptic activity^107^. MAMs at synapses may have a critical role in many aspects of mitochondrial biology, which have a direct impact on synaptic activity. As previously mentioned, the accumulation of Ca^2+^ in the mitochondria leads to the activation of oxidative phosphorylation and to ATP production which is crucial to meet the metabolic demands associated with neuronal activity^111^. However, the sustained mitochondrial Ca^2+^ overload driven by the pacemaking activity in SNpc dopaminergic neurons may ultimately compromise ATP production^93^. Consequently, any types of alteration in ER–mitochondria associations are expected to be potentially damaging to neurons, especially SNpc DA neurons (Fig. 4).

#### α-Synuclein

α-Synuclein has a central role in the pathogenesis of PD^112^, however, the normal function of α-synuclein and its precise role in PD remain poorly understood^113^.

α-Synuclein is a 140 amino acid, lipid-binding protein, which is abundantly expressed in the human, brain and predominantly localized in the presynaptic terminals of neurons. Within neurons, α-synuclein localizes to cytosolic and membrane compartments including synaptic vesicles, mitochondria, and the ER^114,115^. In this regard, its membrane localization involves targeting to lipid rafts, also known as detergent-resistant membranes, enriched in cholesterol and acidic phospholipids^116^. Indeed, a subpopulation of α-synuclein is present in MAM^34,103,117^. Several studies suggest that α-synuclein is involved in modulating synaptic integrity and function^118,119^. In addition, overexpression of wild-type or familial mutant α-synuclein has been shown to damage a plethora of physiological processes. These include Ca^2+^ homeostasis^101,120^, lipid metabolism^103^, the ER^75^, autophagy^121^, and mitochondrial defects^96^. As mentioned previously, all of these physiological processes are regulated by signaling between ER and mitochondria, so the effects of α-synuclein on ER–mitochondria associations have been investigated.

Until now, three different groups have reported that α-synuclein perturbs ER–mitochondria associations^34,101,103^. However, the nature of perturbation differs between these studies.

Cali et al.^101^ reported a role for α-synuclein in modulating ER–mitochondria associations with downstream effects in Ca^2+^ homeostasis in HeLa cells. Indeed, measurement of Ca^2+^ exchange between the two organelles is a recognized measurement of MAM activity^4^. They observed that overexpression of wild-type α-synuclein increases, while downregulation decreases, mitochondrial Ca^2+^ uptake. The quantification of the ER–mitochondria associations also revealed an increase in the co-localization of ER and mitochondrial markers in cells overexpressing wild-type α-synuclein, suggesting that α-synuclein favors ER–mitochondria contacts. Intriguingly, at high levels of α-synuclein expression, induced by high doses of VPA or TAT α-synuclein fusion protein, there was a drastic reduction in mitochondrial Ca^2+^ uptake. The authors observed that at those high levels of overexpression α-synuclein re-localizes into cytoplasmic foci. This may reduce the ability of α-synuclein to mediate ER–mitochondria contacts, representing a loss of function.

Later, Guardia-Laguarta et al.^103^ showed that α-synuclein localizes at MAM and that familial PD mutant α-synuclein associates less than wild-type protein with MAM. This correlates with a decrease in MAM function in cells overexpressing mutant α-synuclein but not wild-type. In this case, the physiological readout of ER–mitochondria associations utilized was the conversion of PS into PE. In these studies, the measurement of ER–mitochondria apposition revealed a lower degree of ER–mitochondria apposition in M17 cells overexpressing familial PD mutant α-synuclein but also in HeLa cells overexpressing the wild-type protein.

Both studies utilized confocal microscopy to analyze ER–mitochondria apposition. However, ER–mitochondria associations are defined by 10–30-nm distances, significantly below of confocal microscopy resolution (∼200 nm)^6,16,122,123^.

Recently, Paillusson et al.^34^ also addressed the role of α-synuclein in ER–mitochondria contacts using high resolution techniques such as electron microscopy, structured illumination microscopy, and proximity ligation assays. Such methods afford better resolution for properly quantifying ER–mitochondria associations. They reported that overexpression of either wild-type or familial PD mutant α-synuclein decreases ER–mitochondria contacts. Consequently, these effects disrupt Ca^2+^ exchange between the two organelles and mitochondrial ATP production. In addition, this study showed that α-synuclein binds to the tethering protein VAPB and decreases the VAPB-PTPIP51 interaction, which is proposed as the mechanism by which it disrupts the contacts. Importantly, this disruption was also seen in neurons derived from induced pluripotent stem cells from familial PD patients harboring pathogenic triplication of the α-synuclein gene^34^.

#### PINK1 and Parkin

Loss-of-function mutations in *PINK1* or *PARK2* genes are associated with juvenile-onset autosomal recessive forms of PD^112^. Parkin (*PARK2* gene expression product) is an ubiquitin E3 ligase that targets specific substrates for degradation. In addition, Parkin has been demonstrated to regulate mitochondrial biogenesis, bioenergetics, dynamics, transport, and degradation^124^. *PINK1* encodes a mitochondrial protein kinase that also protects mitochondrial integrity at different levels. In addition, together with Parkin, PINK1 controls the mechanism of mitophagy^125^. Therefore, upon conditions of mitochondrial depolarization, PINK1 selectively accumulates on the surface of damaged mitochondria, where it phosphorylates and recruits both ubiquitin and Parkin. Parkin then translocates from the cytosol to the OMM and there ubiquitinates specific substrates (such as MFNs and VDAC1), leading their proteasomal degradation^126–128^. Next, these mitochondria are associated to the forming autophagosome membranes by specific ubiquitin-binding receptor proteins (e.g., p62 and optineurin) and afterwards incorporated within autophagosomes^129,130^.

As previously mentioned, several pro-autophagic proteins relocalize to MAMs to initiate autophagosome formation^44,62–64^. Similarly, contact regions between the ER and impaired mitochondria have been shown to be prime locations for Parkin-mediated mitophagy and local recruitment of autophagosome precursors^131^. Recently, Gelmetti et al.^62^ reported that PINK1 and Parkin are recruited to MAM upon mitochondrial depolarization. PINK1 relocation into MAM seems to be necessary for the recruitment of the autophagy machinery to that area. Furthermore, Parkin translocates into ER–mitochondria contact sites in conditions of excitotoxicity in neurons. However in this case, translocation is not associated with mitophagy and it might be instead related with a distinct unknown pathway that needs to be further investigated^104^.

Apart from the aforementioned role in mitophagy, several studies have shown that PARKIN accumulates at both mitochondria and ER–mitochondria associations and modulates ER–mitochondrial crosstalk^102,105^. Once more, the direction of the modulatory effects is controverted.

Cali et al.^102^ reported that Parkin overexpression enhanced ER–mitochondria coupling and its functions. On the contrary, siRNA loss of Parkin caused a decrease in ER–mitochondria signaling associated with weaker mitochondrial Ca^2+^ potentials and ATP production.

Conversely, Gautier et al.^105^ reported that ER–mitochondria associations are instead increased in primary fibroblasts from PARK2 knockout mice and PD patients with PARK2 mutations. This observation correlated with Ca^2+^ dyshomeostasis and increased levels of MFN2^105^.

Once more, these controverted finding highlights the difficulties involved in studies of contact sites between the ER and mitochondria. Morphological changes of these organelles, networks, and technical limitations such as the resolution limits of confocal microscopy, may introduce bias into these analyses. For example, the acute siRNA Parkin depletion used for the first study-induced mitochondrial fragmentation^102^, whereas this is not observed in fibroblasts from PARK2 KO mice or from patients with PARK2 mutations^105^. Another difficulty with these different models is the possibility of potential compensation mechanisms.

#### DJ-1

Diverse mutations, including deletions and point mutations, in the *DJ-1* gene, have been linked to autosomal recessive early-onset parkinsonism^132^. DJ-1 protein has a role in the protection against oxidative stress and mitochondria dynamics; however, the mechanism of its protective function is still unknown. Thus, different functions have been suggested for DJ-1, these include characterization as a redox sensor and an antioxidant scavenger, a chaperone with protease activity, or a transcriptional regulator^133^. DJ-1 is localized in the cytosol and the nucleus. During oxidative stress DJ-1 was shown to translocate to the OMM to maintain a healthy mitochondrial environment^133^. However, Ottolini et al.^106^ showed that DJ-1 was localized at the MAMs but not in the pure mitochondrial fraction. This study also showed that DJ-1 overexpression augmented mitochondrial Ca^2+^ uptake, whereas reduced levels of DJ-1 caused mitochondria fragmentation and decreased mitochondrial Ca^2+^ uptake. By confocal microscopy studies, they also observed an increased ER–mitochondria association when overexpressing DJ-1. Moreover, its overexpression counteracted p53-mediated effects on mitochondrial deregulation, suggesting that DJ-1 might contribute to maintain ER–mitochondria tethering.

## Conclusions and future

Although the exact pathological mechanisms underlying PD remain largely unclear a plethora of cellular pathways are known to be damaged. The discovery that ER–mitochondria signaling, which regulates many of those pathways, are also damaged in PD has highlight the possibility of a common link among them. Therefore, ER–mitochondria signaling may represent a possible drug target upstream of those pathways. However, more research should be done before gaining a clearer understanding of the links between ER–mitochondria signaling and the pathogenesis of PD. Hence, many questions remain unclear. Although the evidence discussed here supports the hypothesis that deregulation of ER–mitochondria signaling has an important role in PD pathogenesis, it is still unclear as to whether ER–mitochondria associations are either upregulated or disrupted upon PD-related insults. Combined, the findings reviewed above highlight the complexity of studying ER–mitochondria associations. Therefore, additional research is needed to gain further insight into the mechanisms of tethering of both organelles, especially in relation to neurons.

Furthermore, investigating whether other PD-related proteins also alter the mitochondria–ER axis or if this is altered in sporadic cases, would be useful to address a possible general pathway for PD. Mutations in LRRK2 are related to both familial and sporadic PD^134^. Autosomal-dominant mutations in LRRK2 have been shown to cause deficits in intracellular Ca^2+^ handling, mitochondrial depolarization and increased mitophagy, which can be prevented by L-type Ca^2+^ channel inhibitors^135–137^. However, whether this is due to altered ER–mitochondria communication remains to be determined.

Another pressing issue is how ER–mitochondria associations can be targeted therapeutically. Likewise, a better understanding in how ER–mitochondria tethers are functionally regulated is crucial to move drug development forward.

In conclusion, more studies are required to enhance our understanding of PD mechanisms and its relation to ER–mitochondria signaling.
